# De novo transcriptome assembly from flower buds of dioecious, gynomonoecious and chemically masculinized female *Coccinia grandis* reveals genes associated with sex expression and modification

**DOI:** 10.1186/s12870-017-1187-z

**Published:** 2017-12-12

**Authors:** Ravi Suresh Devani, Sangram Sinha, Jayeeta Banerjee, Rabindra Kumar Sinha, Abdelhafid Bendahmane, Anjan Kumar Banerjee

**Affiliations:** 1 Biology Division, Indian Institute of Science Education and Research (IISER), Pune, Pune, Maharashtra India; 2 Department of Botany, Tripura University, Suryamaninagar, Tripura India; 3 IPS2, INRA, CNRS, University Paris Sud, University of Evry, University Paris Diderot, University of Paris Saclay, Batiment 630, 91405 Orsay, France

**Keywords:** *Coccinia grandis*, Dioecious, Gynomonoecious, De novo transcriptome, Stamen arrest, Silver nitrate, Ethylene, Pollen fertility

## Abstract

**Background:**

*Coccinia grandis* (ivy gourd), is a dioecious member of Cucurbitaceae having heteromorphic sex chromosomes. Chromosome constitution of male and female plants of *C. grandis* is 22A + XY and 22A + XX respectively. Earlier we showed that a unique gynomonoecious form of *C. grandis* (22A + XX) also exists in nature bearing morphologically hermaphrodite flowers (GyM-H). Additionally, application of silver nitrate (AgNO_3_) on female plants induces stamen development leading to the formation of morphologically hermaphrodite flowers (Ag-H) despite the absence of Y-chromosome. Due to the unavailability of genome sequence and the slow pace at which sex-linked genes are identified, sex expression and modification in *C. grandis* are not well understood.

**Results:**

We have carried out a comprehensive RNA-Seq study from early-staged male, female, GyM-H, and Ag-H as well as middle-staged male and GyM-H flower buds. A de novo transcriptome was assembled using Trinity and annotated by BLAST2GO and Trinotate pipelines. The assembled transcriptome consisted of 467,233 ‘Trinity Transcripts’ clustering into 378,860 ‘Trinity Genes’. Female_Early_vs_Male_Early, Ag_Early_vs_Female_Early, and GyM-H_Middle_vs_Male_Middle comparisons exhibited 35,694, 3574, and 14,954 differentially expressed transcripts respectively. Further, qRT-PCR analysis of selected candidate genes validated digital gene expression profiling results. Interestingly, ethylene response-related genes were found to be upregulated in female buds compared to male buds. Also, we observed that AgNO_3_ treatment suppressed ethylene responses in Ag-H flowers by downregulation of ethylene-responsive transcription factors leading to stamen development. Further, GO terms related to stamen development were enriched in early-staged male, GyM-H, and Ag-H buds compared to female buds supporting the fact that stamen growth gets arrested in female flowers.

**Conclusions:**

Suppression of ethylene responses in both male and Ag-H compared to female buds suggests a probable role of ethylene in stamen suppression similar to monoecious cucurbits such as melon and cucumber. Also, pollen fertility associated GO terms were depleted in middle-staged GyM-H buds compared to male buds indicating the necessity of Y-chromosome for pollen fertility. Overall, this study would enable identification of new sex-biased genes for further investigation of stamen arrest, pollen fertility, and AgNO_3_-mediated sex modification.

**Electronic supplementary material:**

The online version of this article (10.1186/s12870-017-1187-z) contains supplementary material, which is available to authorized users.

## Background

Monoecy, dioecy, and hermaphroditism are the three major sexual forms observed among the flowering plants. Ninety (90%) of angiosperms are found to be hermaphrodite (both male and female organs are in the same flower), while 5% plant species exhibit monoecy (male and female flowers are on the same plant) and remaining 5% show dioecy (male and female flowers are in separate plant) [1]. Dioecism provides a unique opportunity to study the genetic basis of sex determination. *Silene latifolia* (Caryophyllaceae), *Rumex acetosa* (Polygonaceae), *Carica papaya* (Caricaceae), *Spinacia oleracea* (Chenopodiaceae) and *Populus* (Salicaceae), have been well characterized to understand the mechanism of sex determination [2–5]. However, the molecular mechanism and the genes that govern sex determination are not well understood.

*Coccinia grandis* (L.) Voigt, a dioecious member of Cucurbitaceae family having an inferior ovary has received comparatively less attention. Members of Cucurbitaceae family exhibit variety of sexual forms [6]. Apart from its rich medicinal value, C. *grandis*, commonly known as ivy gourd, is also used as a vegetable. *Coccinia grandis* bears male and female unisexual flowers on separate plants. Similar to *Silene latifolia* (Caryophyllaceae), the sex in *Coccinia grandis* is determined by the presence of Y-chromosome [7–9]. The chromosome constitution of male and female plants is 22A + XY and 22A + XX respectively, where Y-chromosome is larger than the X-chromosome [10–12]. The male flower consists of three convoluted (bithecous) stamens [13, 14] and lacks female reproductive organs; however, the female flower consists of three rudimentary stamens surrounding the three fused carpels with an inferior ovary [14]. There are two ways by which unisexual flower development can be achieved. One of the ways is when both male and female sex organ primordia are initiated at early stages of flower development, but at later stages the opposite sex organs are aborted as in *Silene latifolia* [15]*.* Another way is that organ primordia of the opposite sex organs do not develop at all as shown in *Thalictrum dioicum* [16]*.* Also, there are flowers, wherein the inappropriate sex organs are retained in rudimentary form (instead of getting aborted) as in *Rumex* and *C. grandis* [2, 14]. Additionally, *Coccinia grandis* shows sex modification upon application of AgNO_3_ leading to the development of stamens in female flower (such flower will be referred to as Ag-H) as described in our previous report [14]. Ag^+^ has been long known to be an inhibitor of ethylene response [17]. It has been suggested that the binding of Ag^+^ to the ethylene receptor inhibits the conformational change, which maintains the receptor in the active conformation [18]. Application of silver compounds such as silver nitrate (AgNO_3_) or silver thiosulphate (Ag_2_S_2_O_3_) masculinizes monoecious plants such as *Cucumis sativus* as well as female plants of dioecious species such as *Silene latifolia* and *Cannabis sativa* [19–21]. However, the mechanism of action by which Ag^+^ induces stamen development is not known till date [21].

Despite the interesting discovery of sex chromosomes in dioecious plants more than 50 years ago, the mechanism of sex determination remains poorly understood [22, 23]. This is primarily because of the slow pace at which sex-linked genes were identified from dioecious species (one to two genes/year) [24]. However, the improvement in NGS technology has already started changing the situation by accelerating the rate of sex-linked gene identification. The NGS-based approach has a big advantage that it does not require prior knowledge of the gene sequences to be investigated. Recently, an NGS-based RNA-Seq approach was applied to *Silene latifolia,* which was the first report that demonstrated the phenomenon of dosage compensation in plants [24]. A comparative transcriptomics approach was applied to papaya, a trioecious species, to identify the candidate genes for sex determination. This study led to the identification of 312 unique tags that were specifically mapped to the primitive sex chromosome (X or Y^h^) sequences in papaya [5]. A genome-wide transcriptional profiling of apical tissue of a gynoecious mutant (Csg-G) and the monoecious wild-type (Csg-M) of cucumber was also performed to isolate genes involved in sex determination. This study revealed that genes involved in plant hormone signaling pathways, such as *ACS*, *Asr1*, *CsIAA2*, *CS-AUX1*, and *TLP*, and their crosstalk might play a critical role in the sex determination. Authors have also predicted the regulation of some transcription factors, including *EREBP*-9, in sex determination [25]. In another study, transcriptome sequencing was carried out from cucumber flower buds of two near-isogenic lines, WI1983G, a gynoecious plant which bears only pistillate flowers and WI1983H, a hermaphroditic plant which bears only bisexual flowers [26]. This study identified differentially expressed genes as well as putative SSR and SNP markers between flowers of two different sexes. T Akagi, IM Henry, R Tao and L Comai [27] sequenced genomic DNA, mRNA as well as small RNA from flower buds of persimmon and identified a Y-chromosome–encoded small RNA, *OGI,* that targets a homeodomain transcription factor *MeGI* regulating pollen fertility in a dosage-dependent manner. A recent de novo transcriptomics study in garden *Asparagus* identified 570 differentially expressed genes, where genes involved in pollen microspore and tapetum development were shown to be specifically expressed in males and supermales in contrast to females [28].

In order to identify sex-biased genes in *C. grandis* and to elucidate the mechanism of AgNO_3_ mediated sex modification, a comprehensive RNA-Seq study from early-staged male (M), female (F), GyM-H and Ag-H as well as middle-staged male and GyM-H flower buds was carried out. De novo transcriptome was assembled to identify *C. grandis* homologs of various flower development genes. Digital expression profiling was undertaken to identify sex-biased genes which might play a pivotal role in the arrest of stamen development in female flowers, genes that promote anther development in female flowers upon AgNO_3_ treatment and genes controlling pollen fertility in male flowers.

## Methods

### Flower bud collection and RNA isolation

**Fig. 1:**
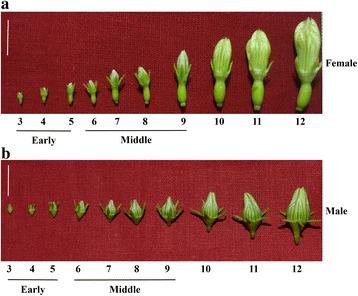
Different stages of *C. grandis* flower buds selected for RNA-Seq analysis. (**a**) Male, (**b**) Female flower buds. GyM-H and Ag-H buds sized similar to female buds were chosen for RNA seq study as described in our previous work (Ghadge et al., 2014). Scale bar = 1 cm

Clones of wild-type male, female and gynomonoecious (GyM) forms of *C. grandis* were grown in the experimental plot at IISER Pune, India. Gynomonoecious (GyM) plant bears pistillate flowers (GyM-F) as well as morphologically hermaphrodite flowers (GyM-H) (Herbarium Voucher: Tripura University Campus, Karmakar, 433). Foliar spray of 35 mM AgNO_3_ solution to the basal leaves on some of the female plants led to the development of morphologically hermaphrodite flowers (Ag-H) as per our earlier observation [14]. Ag-H flower buds were morphologically similar to GyM-H flowers. Flower buds from male (M), female (F), GyM-H and Ag-H were harvested separately in liquid nitrogen and categorized into early and middle stages based on our previous study [14] (Fig. 1). In early-staged male flower buds, only stamens are present with no sign of carpel initials. Whereas, early-staged female flowers (stages 3–4, Fig. 1) have both carpel and stamen primordia. Stamen growth in female flowers gets arrested around stages 4–5. In the hermaphrodite flowers of gynomonoecious plant, however, both stamens and carpels develop simultaneously during early as well as middle stages of development. Our selection of early-staged flower buds was carried out such that the event of stamen inhibition in female flowers can be analysed. Whereas, middle-staged flower buds were chosen such that meiosis-stage and pollen maturation event can be investigated. Total RNA was isolated by TRIzol reagent (Invitrogen) following the manufacturer’s instructions. RNA quality was assessed using an Agilent Bioanalyzer RNA nanochip, and RNA samples with RIN > 8.0 were used for library preparation.

### RNA-Seq library preparation and sequencing

Library preparation was performed at Genotypic Technology’s Genomics facility, Bangalore using Illumina TruSeq RNA Sample Preparation Kit according to the manufacturer’s specifications. RNA sequencing libraries were prepared in duplicate for early-staged male (M), female (F), GyM-H and Ag-H flower buds, as well as middle-staged male (M) and GyM-H flower buds. The quality of all the twelve libraries and insert size distribution was assessed using an Agilent High Sensitivity Bioanalyzer Chip. Libraries showed a peak in the range of 250–1000 bp. The effective sequencing insert size was 130–880 bp, and the inserts were flanked by adaptors whose combined size was 120 bp. Libraries were quantified using Qubit and sequenced on Illumina NextSeq 500 platform, producing 2 X 150-nucleotide paired-end reads. RNA-Seq data generated in this study has been deposited in the NCBI SRA study SRP111347.

### Pre-processing of Illumina reads and de novo transcriptome assembly

Raw RNA-Seq reads were processed using Trimmomatic v0.33 for trimming adapters as well as low-quality bases from ends of the reads [29]. Poor quality reads with average Phred quality score < 20 and reads with length < 36 were also filtered out. The resulting set of good quality reads were then assembled with Trinity v2.1.1 software using default parameters [30, 31].

The quality of the resulting assembly was assessed by various methods. First of all, RNA-Seq read representation of the assembly was checked using bowtie2 [32]. Ex90N50 transcript contig length (the contig N50 value based on the set of transcripts representing 90% of the expression data) was computed using *contig_ExN50_statistic.pl* script bundled with Trinity. Then the representation of full-length reconstructed protein-coding genes was studied. The assembled transcripts were compared with Swiss-Prot using BLAST and the hits were analyzed using a perl script *blast_outfmt6_group_segments.tophit_coverage.pl,* provided with the Trinity package. BUSCO (Benchmarking Universal Single-Copy Orthologs) was used to explore completeness of the transcriptome according to conserved ortholog content [33]. Finally, TransRate was used to compare the assembly to the publicly available *Cucumis sativus* protein-coding primary transcript sequences [34].

### Annotation of the de novo-assembled transcripts

The de novo-assembled transcripts were compared with the viridiplantae sequences from nr and Swiss-Prot database using BLASTX with an e-value threshold of 1e-3 [35]. BLAST output generated from this comparison was loaded into BLAST2GO for mapping GO terms to the transcripts and annotation [36]. Enzyme codes and KEGG pathway mapping were also carried out. ANNEX (Annotation Expander) was used to enhance the annotations. Finally, GO-Slim mapping was applied to get a broad overview of the ontology content.

In addition to BLAST2GO facilitated annotation, Trinotate pipeline was used to carry out comprehensive functional annotation of the transcripts leveraging various annotation databases (eggNOG/GO/KEGG databases) [31]. Trinotate pipeline also included identification of open reading frames, homology search against Swiss-Prot and TrEMBL. Protein domain identification was carried out using HMMER/PFAM. Protein signal peptide and transmembrane domains were predicted by signalP and tmHMM respectively.

### Transcript quantification and differential expression analysis

*align_and_estimate_abundance.pl* script from Trinity package was applied to align cleaned reads from each library to the de novo transcriptome using bowtie and to estimate the transcript abundance using RSEM [37]. *abundance_estimates_to_matrix.pl* script was used to construct a matrix of counts and a matrix of normalized expression values. *PtR* script was used to generate correlation matrix and Principal Component Analysis (PCA) plot for comparing replicates across all the samples. Differential expression analysis was carried out with two biological replicates from the count matrix using *run_DE_analysis.pl* with edgeR as the method of choice [38]. *analyze_diff_expr.pl* script was used to examine GO enrichment and to extract all transcripts that had *p*-values at most 1e-3 and were at least 2^2 fold differentially expressed. The DE features were partitioned into clusters with similar expression patterns by *define_clusters_by_cutting_tree.pl* script with Ptree method.

### Validation of differentially expressed genes by qRT-PCR

For expression analysis, qRT-PCR was carried out using aliquots of the same RNA samples that were used for RNA sequencing. Two micrograms (2 μg) of total RNA was used for complementary DNA (cDNA) synthesis by SuperScript IV reverse transcriptase (Invitrogen) using an oligo(dT) primer. *CgACT2* gene was used as reference gene for normalization. qRT-PCR was performed on BIO-RAD CFX96 machine with gene-specific forward and reverse primers (Additional file 1: Table S1). The reactions were carried out using Takara SYBR Premix Ex Taq II (Takara Bio Inc.) and incubated at 95 °C for 3 min followed by 40 cycles of 95 °C for 15 s, 58 °C for 15 s and 72 °C for 15 s. PCR specificity was checked by melting curve analysis, and data were analysed using the 2^–∆∆CT^ method [39].

## Results

### RNA sequencing, Trinity-based de novo transcriptome assembly and annotation using BLAST2GO and Trinotate

**Fig. 2:**
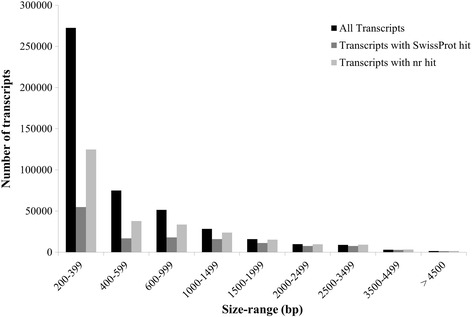
Transcript size distribution for *C. grandis* flower bud transcriptome

A total of 306,575,536 paired-end reads (150 bp) were obtained after sequencing all the twelve libraries on the Illumina NextSeq 500 platform. Subsequently, 186,399,131 good quality paired-end reads were used for de novo assembly of *Coccinia grandis* flower bud transcriptome using Trinity software package with default parameters (Table 1). The resulting assembly consisted of 467,233 ‘Trinity Transcripts’ clustering into 378,860 ‘Trinity Genes’ with an N50 value of 881 bp (Table 2, Additional file 2). The transcripts of 200–399 bp size were found to be most abundant in the length distribution of assembled transcripts (Fig. 2). However, a higher proportion of transcripts with length around 1000–2000 bp had a BLAST hit compared to the proportion of smaller transcripts (Fig. 2). Cleaned reads were mapped back to the transcriptome using bowtie2 with ~70% or more reads from each library aligning concordantly (Table 1). An Ex90N50 statistic calculated using 80,806 transcripts from the assembly (ignoring the rest of the transcripts with poor read coverage) was found to be 1784 bp (Additional file 3: Figure S1).

**Table 1:** RNA sequencing read counts and alignment statistics for all the samples used for de novo transcriptome assembly

Sample name	Raw Reads	Cleaned reads	% Read pairs mapped concordantly
Male Early A	21,719,110	13,216,446	76.33%
Male Early B	22,080,977	12,149,633	74.84%
Female Early A	25,607,195	15,634,598	76.34%
Female Early B	25,433,955	15,316,996	75.94%
GyM-H Early A	27,936,206	17,378,706	76.67%
GyM-H Early B	27,617,808	17,107,330	76.69%
Ag-H Early A	26,147,527	15,140,736	71.02%
Ag-H Early B	25,392,540	15,128,539	69.35%
Male Middle A	25,502,209	15,490,027	76.70%
Male Middle B	25,837,400	15,919,727	75.27%
GyM-H Middle A	28,465,770	17,441,923	76.90%
GyM-H Middle B	24,834,839	16,474,470	77.43%
Total	306,575,536	186,399,131

**Table 2:** Assembly statistics for *C. grandis* flower bud transcriptome

Parameter	Assembly statistics
Number of ‘Trinity Transcripts’	467,233
Number of ‘Trinity Genes’	378,860
Percent GC	38.96
Median contig length (bp)	347
Average contig length (bp)	606.45
N50 (bp)	881
Total assembled bases	283,354,298

Altogether, 8916 unique BLAST hits in the Swiss-Prot database were represented by nearly full-length transcripts, having more than 70% alignment coverage, and 12,315 hits showed more than 50% alignment coverage (Additional file 4: Table S2). BUSCO output [C:89.8%(S:14.5%,D:75.3%),F:5.0%,M:5.2%,n:1440] showed that out of 1440 BUSCOs for Plants dataset, 1293 full length BUSCOs were detected in our de novo-assembled *Coccinia grandis* flower bud transcriptome indicating 89.8% completeness. Finally using TransRate, we were able to detect *C. grandis* homologs for 84% (18,039) of protein-coding primary transcripts of *C. sativus* of which, 13,430 reference sequences had at least 95% of their bases covered by a CRB-BLAST hit (Additional file 5: Table S3).

**Fig. 3:**
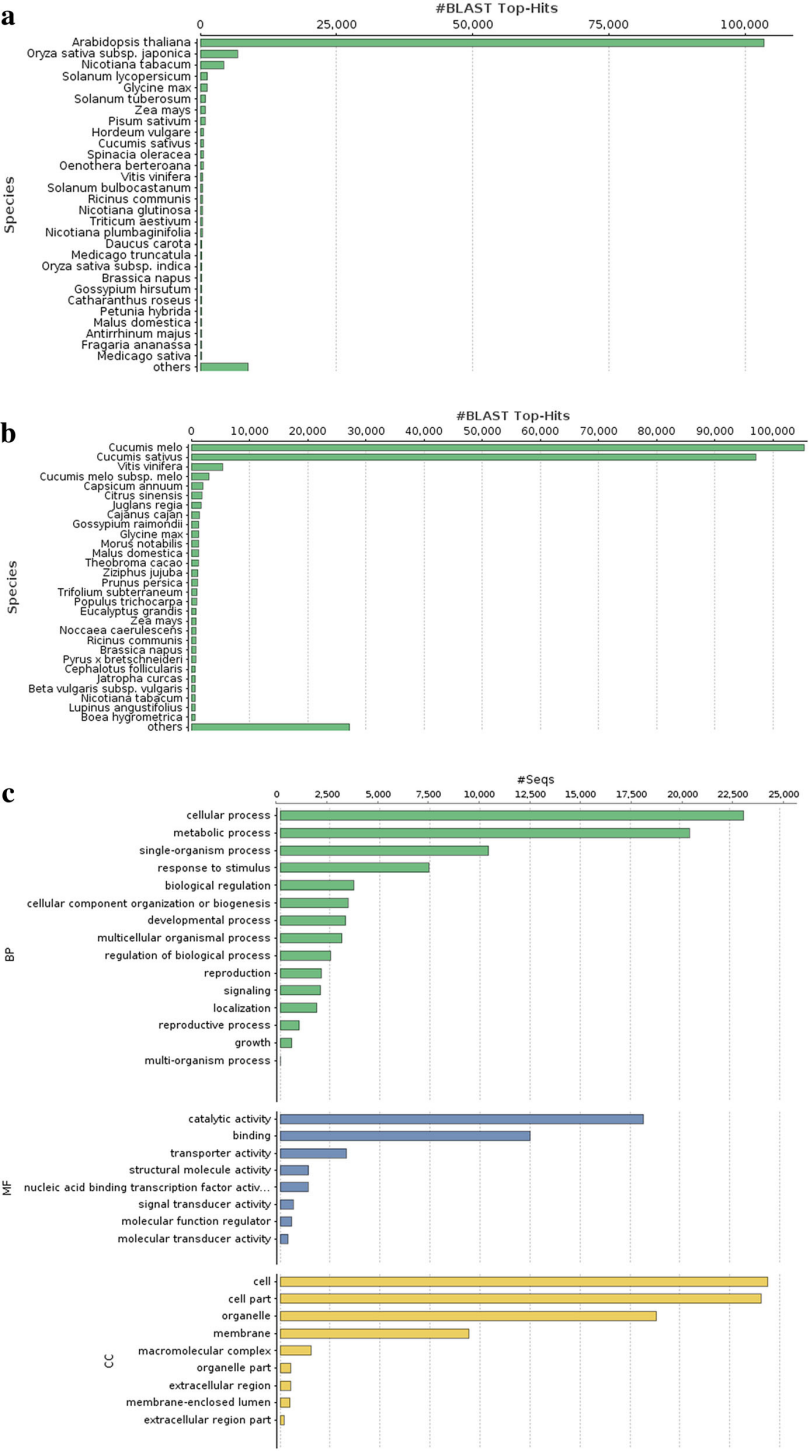
BLAST2GO annotation of *C. grandis* flower bud transcriptome. (**a**) BLAST Top-Hits species distribution when compared with Swiss-Prot database, (**b**) BLAST Top-Hits species distribution when compared with nr database, (**c**) GO category distribution of *C. grandis* flower bud transcriptome

*Coccinia grandis* flower bud transcripts were compared to plant protein sequences of the nr and Swiss-Prot databases resulting in 259,200 and 136,663 transcripts having at least one hit from the respective database. Species distribution analysis of the BLAST hits showed that majority of these hits were from *Arabidopsis* and rice for Swiss-Prot database whereas for nr database most top hits were from cucumber and melon (Fig. 3a,b). The number of transcripts annotated with various GO terms of biological process, molecular function, and cellular component categories are provided in Fig. 3c.

Trinotate v3 pipeline was also used simultaneously for comprehensive functional annotation of the *Coccinia grandis* flower bud transcripts. Details regarding the Swiss-Prot/TrEMBL BLAST hits, GO, KEGG and eggNOG mappings can be found in the Additional file 6: Table S4. HMMER/PFAM predicted protein domains, as well as information regarding signal peptides and transmembrane domains could also be found in the Additional file 6: Table S4. Taken together, we have assembled a good quality transcriptome for early- and middle-staged flower buds of *Coccinia grandis* and comprehensively annotated the transcripts using well-established BLAST2GO and Trinotate pipelines.

### Differential expression analysis reveals probable factors for pollen fertility and sex modification

**Fig. 4:**
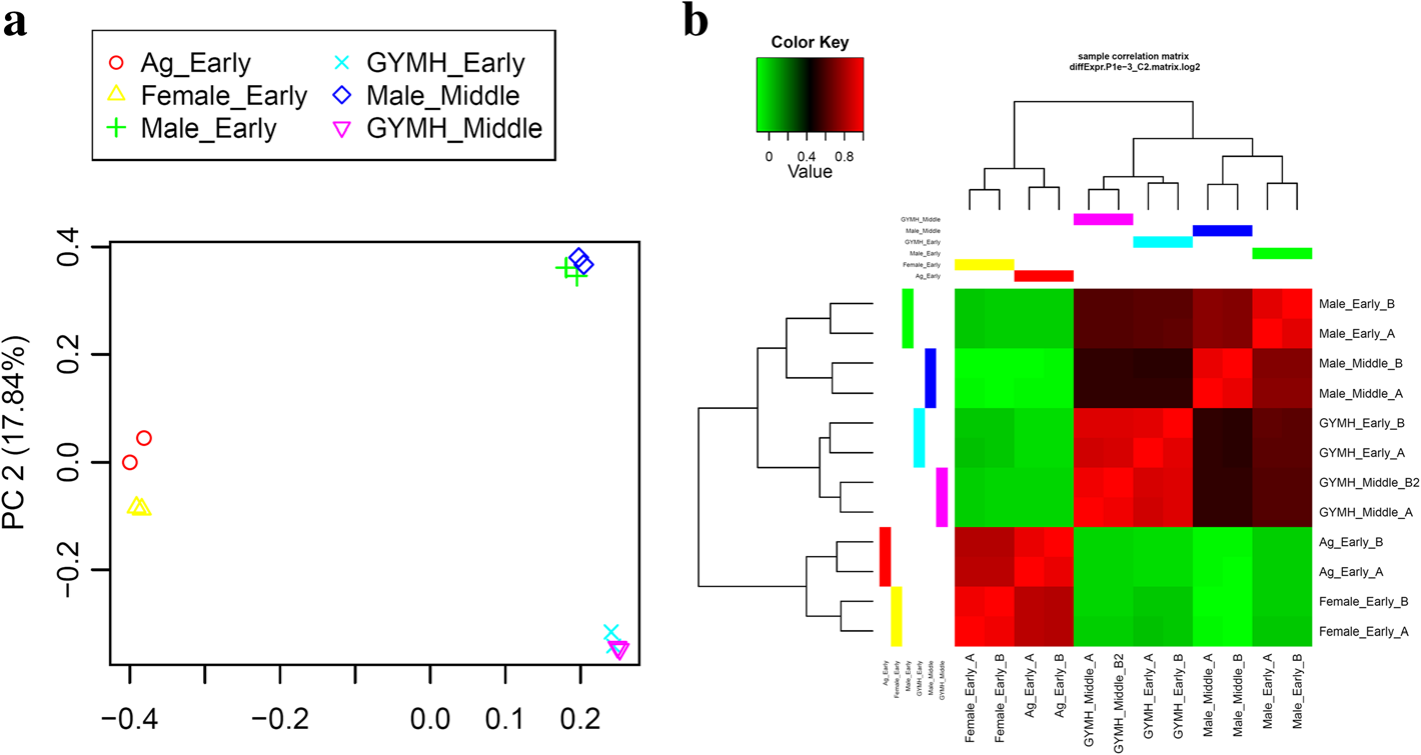
Correlation analyses showing the relationship between samples and replicates. (**a**) Principal component analysis and (**b**) correlation matrix showing relationship between all samples as well as replicates

**Fig. 5:**
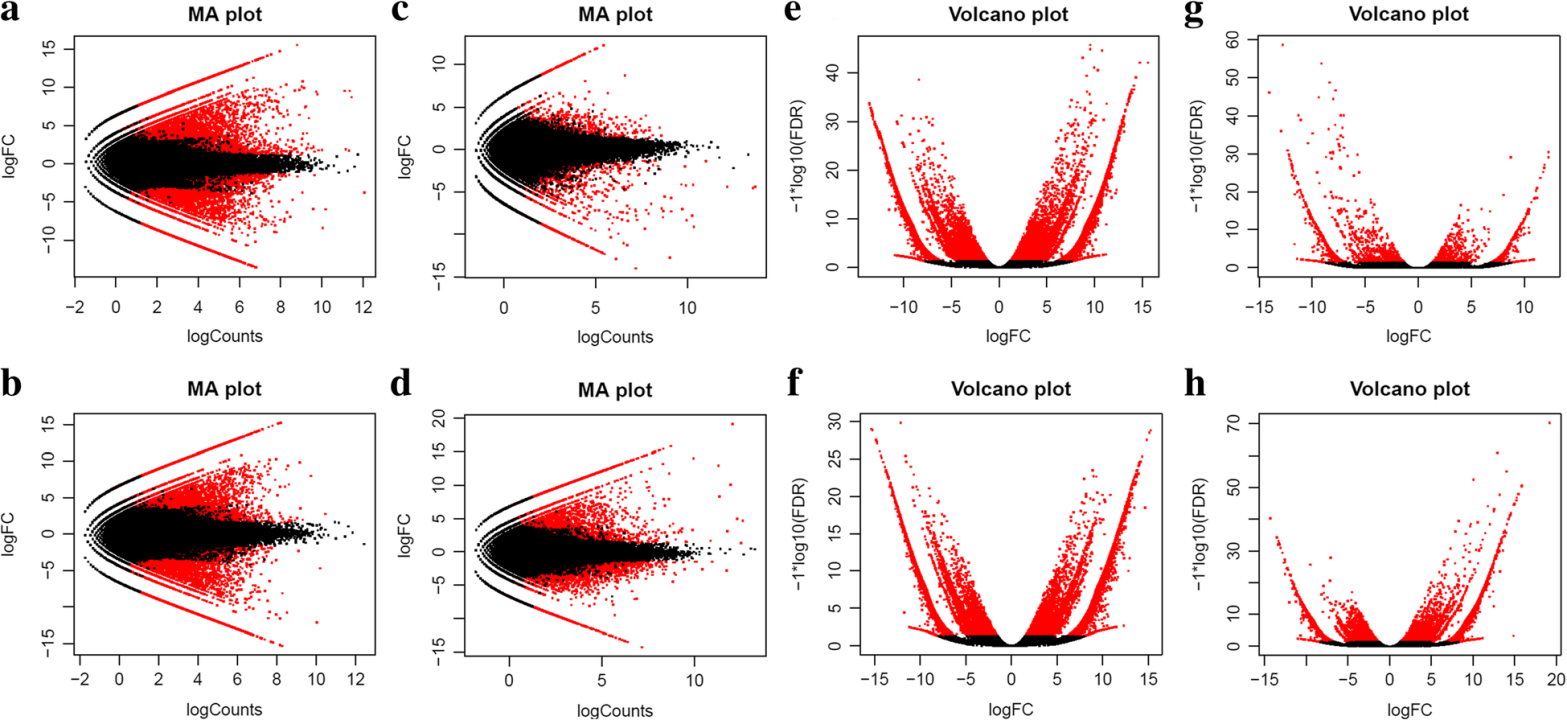
Pairwise comparisons of transcript abundance. MA plots showing average log fold change (logFC) vs average log of counts among (**a**) female (early-staged) vs. male (early-staged) transcripts, (**b**) female (early-staged) vs. GyM-H (early-staged) transcripts, (**c**) Ag-H (early-staged) vs. female (early-staged) transcripts and (**d**) GyM-H (middle-staged) vs. male (middle-staged) across replicates. Volcano plots showing differentially expressed transcripts in relation to FDR (False discovery rate) for (**e**) female (early-staged) vs. male (early-staged) transcripts, (**f**) female (early-staged) vs. GyM-H (early-staged) transcripts, (**g**) Ag-H (early-staged) vs. female (early-staged) transcripts and (**h**) GyM-H (middle-staged) vs. male (middle-staged). Features found DE at FDR <0.05 are colored red. Features with *P*-values at most 1e-3 and at least 2^2 fold change are differentially expressed

**Fig. 6:**
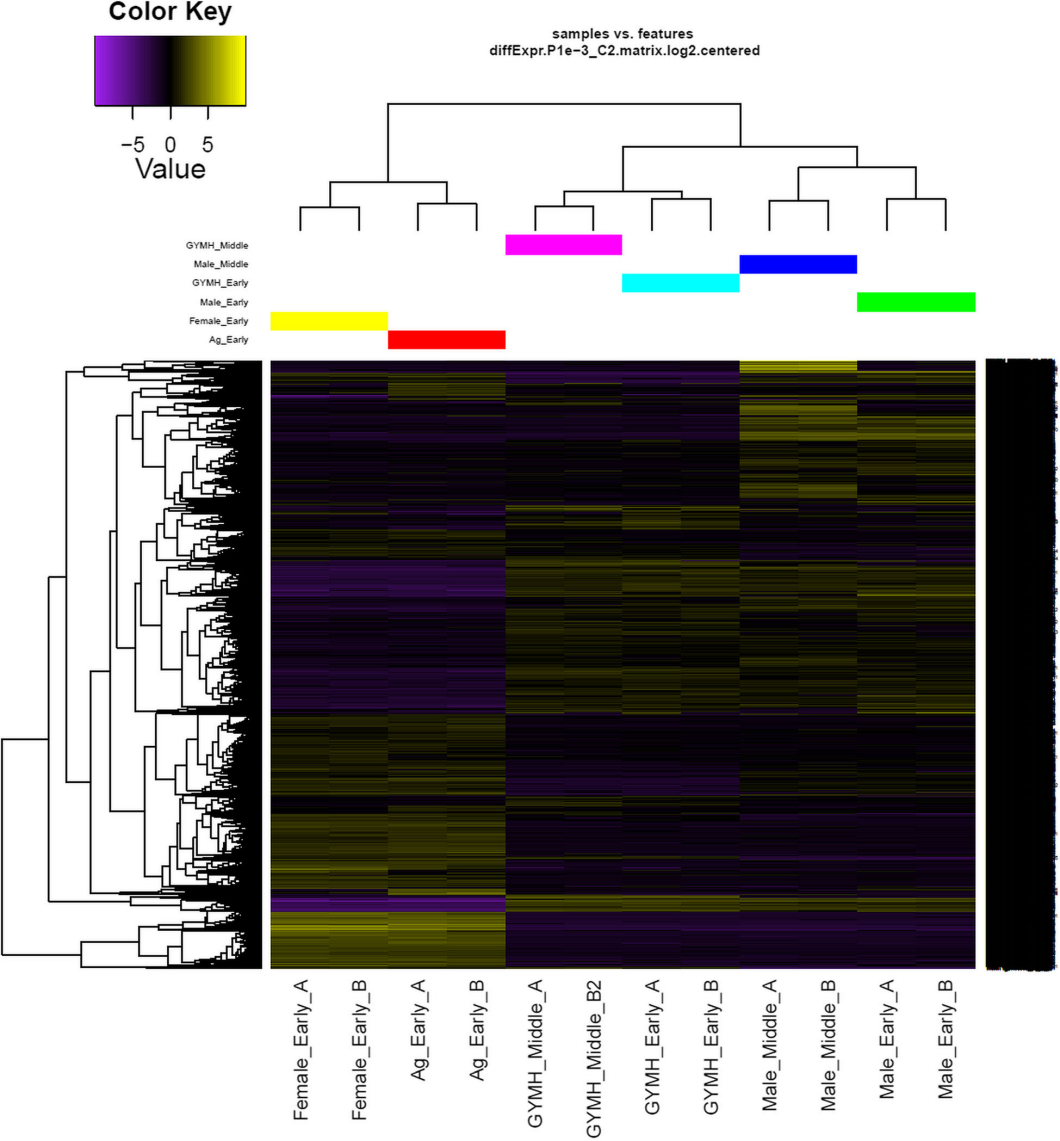
Hierarchical clustering of differentially expressed transcripts and developmentally staged *C. grandis* flower bud samples. Heatmap showing the relative expression levels of each transcript (rows) in each sample (columns). Rows and columns are hierarchically clustered. Expression values (FPKM) are log_2_ –transformed and then median-centered by transcript

**Fig. 7:**
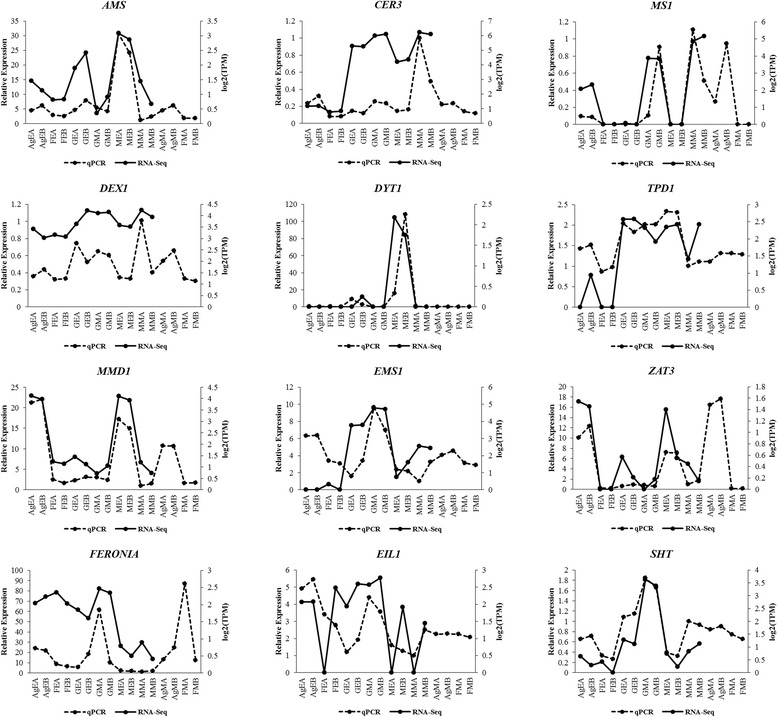
Validation of selected DE genes by qRT-PCR with two biological replicates. The relative expression in the sample of middle-staged male-A (MMA) was set to 1 for plotting the qRT-PCR data. AgEA, Early-staged Ag-H A; AgEB, Early-staged Ag-H B; FEA, Early-staged Female A; FEB, Early-staged Female B; GEA, Early-staged GyM-H A; GEB, Early-staged GyM-H B; GMA, Middle-staged GyM-H A; GMB, Middle-staged GyM-H B; MEA, Early-staged Male A; MEB, Early-staged Male B; MMA, Middle-staged Male A; MMB, Middle-staged Male B; AgMA, Middle-staged Ag-H A, AgMB, Middle-staged Ag-H B; FMA, Middle-staged Female A; FMB, Middle-staged Female B

**Fig. 8:**
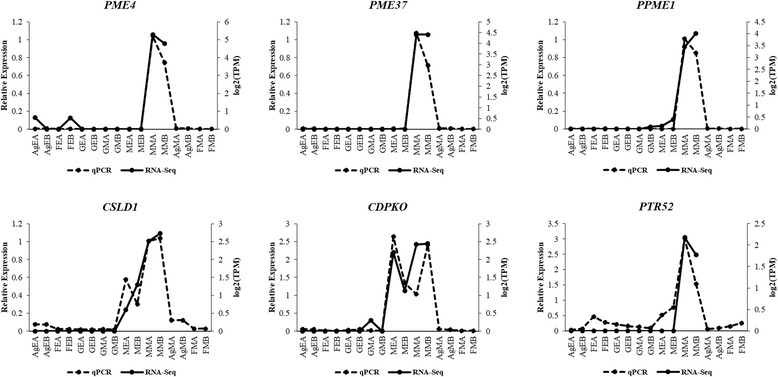
qRT-PCR based expression analyses of selected genes involved in pollen tube development with two biological replicates. The relative expression in the sample of middle-staged male-A (MMA) was set to 1 for plotting the qRT-PCR data. AgEA, Early-staged Ag-H A; AgEB, Early-staged Ag-H B; FEA, Early-staged Female A; FEB, Early-staged Female B; GEA, Early-staged GyM-H A; GEB, Early-staged GyM-H B; GMA, Middle-staged GyM-H A; GMB, Middle-staged GyM-H B; MEA, Early-staged Male A; MEB, Early-staged Male B; MMA, Middle-staged Male A; MMB, Middle-staged Male B; AgMA, Middle-staged Ag-H A, AgMB, Middle-staged Ag-H B; FMA, Middle-staged Female A; FMB, Middle-staged Female B

**Fig. 9:**
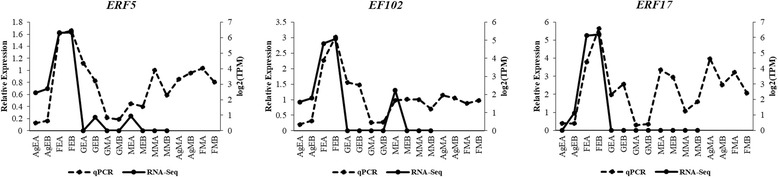
qRT-PCR based expression analyses of selected *Ethylene-responsive transcription factors* (*ERFs*) with two biological replicates. The relative expression in the sample of middle-staged male-A (MMA) was set to 1 for plotting the qRT-PCR data. AgEA, Early-staged Ag-H A; AgEB, Early-staged Ag-H B; FEA, Early-staged Female A; FEB, Early-staged Female B; GEA, Early-staged GyM-H A; GEB, Early-staged GyM-H B; GMA, Middle-staged GyM-H A; GMB, Middle-staged GyM-H B; MEA, Early-staged Male A; MEB, Early-staged Male B; MMA, Middle-staged Male A; MMB, Middle-staged Male B; AgMA, Middle-staged Ag-H A, AgMB, Middle-staged Ag-H B; FMA, Middle-staged Female A; FMB, Middle-staged Female B

Following transcriptome assembly and annotation, differential expression analysis was carried out. First of all, RSEM was used for transcript abundance estimation. Following which, we checked for the correlation between the replicates for all the samples using *PtR* script. PCA analysis and correlation matrix showed a good correlation between the replicate sets for each of the six samples (Fig. 4). EdgeR was used to identify the differentially expressed transcripts for all the pairwise comparisons between the six samples (Table 3; Fig. 5; Additional file 7: Figure S2). Differentially expressed transcripts at a minimum fold change of 2^2 with *p*-values at most 1e-3 were extracted and GO enrichment analysis was performed (Additional file 8: Table S5, Additional file 9: Table S6). Among all the comparisons, few interesting ones such as Ag_Early_vs_Female_Early, Female_Early_vs_Male_Early, and GYM-H_Middle_vs_Male_Middle had 3574, 35,694 and 14,954 differentially expressed transcripts respectively (Table 3, Fig. 5). The DE features were partitioned into clusters with similar expression patterns (Fig. 6; Additional file 10: Figure S3). In the context of anther development, we identified several GO terms (GO:0080110, GO:0010208, GO:0010584, GO:0009555, GO:0055046, GO:0048658, GO:0048653) differentially enriched in male buds compared to female buds at an early stage of floral development. qRT-PCR was done to validate the results of differential expression analysis for a few interesting *Coccinia* homologs of *AMS* (ABORTED MICROSPORES), *CER3* (ECERIFERUM 3), *DEX1* (DEFECTIVE IN EXINE FORMATION 1), *DYT1* (DYSFUNCTIONAL TAPETUM 1), *EIL1* (ETHYLENE INSENSITIVE 3-like 1), *EMS1* (EXCESS MICROSPOROCYTES 1), *FER* (FERONIA), *MMD1* (MALE MEIOCYTE DEATH 1), *MS1* (MALE STERILITY 1), *SHT* (Spermidine hydroxycinnamoyl transferase), *TPD1* (TAPETUM DETERMINANT 1) and *ZAT3* (Zinc finger protein ZAT3). Expression profiles for these genes deduced by qRT-PCR revealed similar patterns to that seen from the digital DE analysis results (Fig. 7). Also, we have found GO terms related to pollen fertility enriched in the male buds (GO:0080092, GO:0009846, GO:0009860) compared to GyM-H and Ag-H buds, which had sterile pollens. Accordingly, expression profile for homologs of a number of genes involved in pollen tube development such as *CSLD1* (Cellulose synthase-like protein D1; TRINITY_DN92683_c0_g1_i1), *CDPKO* (Calcium-dependent protein kinase 24; TRINITY_DN93671_c0_g1_i3), *PME4* (Pectin methylesterase 4; TRINITY_DN14239_c0_g1_i1), *PME37* (Pectin methylesterase 37; TRINITY_DN3663_c0_g1_i1), *PPME1* (POLLEN SPECIFIC Pectin methylesterase 1; TRINITY_DN66415_c0_g1_i1) and *PTR52* (Protein NRT1/PTR FAMILY 2.8; TRINITY_DN112735_c0_g14_i3) were analysed and found to be enriched in middle-staged male buds similar to our digital expression profiles based on RNA-Seq data (Fig. 8). Downregulation of ethylene signalling upon AgNO_3_ treatment was evident as GO:0009723 (response to ethylene) and GO:0009873 (ethylene-activated signaling pathway) were depleted in AgNO_3_ treated plant (Additional file 9: Table S6). In order to validate this, we studied the expression profile of *Ethylene-responsive transcription factors*, *ERF5*, *ERF17,* and *EF102*. We found that all three *ERFs* were downregulated upon AgNO_3_ treatment (Fig. 9).

**Table 3:** Number of differentially expressed transcripts for each pairwise comparison between the flower types. Transcripts that had p-values at most 1e-3 and were at least 2^2 fold were considered as differentially expressed

Flower Buds Comparison	Number of DE transcripts
Ag_Early_vs_Female_Early	3574
Ag_Early_vs_GYMH_Early	34,458
Ag_Early_vs_GYMH_Middle	38,849
Ag_Early_vs_Male_Early	33,863
Ag_Early_vs_Male_Middle	36,923
Female_Early_vs_GYMH_Early	31,886
Female_Early_vs_GYMH_Middle	39,885
Female_Early_vs_Male_Early	35,694
Female_Early_vs_Male_Middle	40,477
GYMH_Early_vs_GYMH_Middle	816
GYMH_Early_vs_Male_Early	8659
GYMH_Early_vs_Male_Middle	11,576
GYMH_Middle_vs_Male_Early	12,357
GYMH_Middle_vs_Male_Middle	14,954
Male_Early_vs_Male_Middle	4427

## Discussion

Genetic basis of sex determination and differentiation is not well studied in *C. grandis*. Identification and investigation of sex-linked genes would lead to better understanding of dioecy in plants and this can be achieved by whole genome sequencing approach. However, sex-determining genes are most likely linked to non-recombining regions of Y-chromosome, which are difficult to assemble from sequence data [40]. An alternative approach is to use comparative transcriptomics to identify sex-biased genes that could play a role in sex differentiation and determination [24]. Further, the presence of mutations and SNPs in sex-biased genes can provide insights regarding the evolution of dioecy. Using this approach, we have assembled and annotated a de novo transcriptome from the flower buds of dioecious, gynomonoecious and AgNO_3_ treated female *C. grandis.* We have identified differentially expressed genes which might be playing a role in stamen arrest of female flowers. Also, we have analysed the genes that were differentially expressed upon AgNO_3_ treatment on female plants promoting stamen development. Finally, we have compared middle-staged male (bearing fertile pollens) and GyM-H buds (bearing sterile pollens) to study the genes involved in pollen maturation and fertility.

### Differential expression of stamen developmental genes and arrest of stamen growth in female flowers

At the early stages (stages 3–4) of flower development in female *C. grandis,* both carpel and stamen organs are initiated simultaneously. However, stamen growth gets arrested during the course of development (stages 4–5) resulting in a female flower with rudimentary stamens. In contrast, no sign of carpel primordia was observed during the histological study of flower development in male *C. grandis* as described in our previous report [14]. The molecular players involved in stamen initiation and development process are well characterized in the hermaphrodite plant *Arabidopsis*. In order to identify the stage at which stamen growth gets arrested, *Coccinia grandis* homologs of *Arabidopsis* stamen development genes were identified from the de novo-assembled transcriptome. Among genes involved in stamen initiation, *Pistillata* (*CgPI*, TRINITY_DN71631_c0_g1_i1) was found to be expressed in a male-biased fashion (Additional file 8: Table S5). *Pistillata* has been shown to specify stamen identity in *Arabidopsis* [41] (Table 4). Further, EXCESS MICROSPOROCYTES 1 (EMS1) has been shown to interact with TAPETUM DETERMINANT 1 (TPD1) regulating specification of reproductive as well as somatic cells in *Arabidopsis* anthers [42]. Differential expression analyses revealed that homologs of both *EMS1* (TRINITY_DN106236_c0_g4_i1) and *TPD1* (TRINITY_DN116795_c2_g1_i3) were enriched in male flowers compared to female flowers (Table 4; Additional file 8: Table S5; Fig. 7). *DYSFUNCTIONAL TAPETUM 1* (*DYT1)* plays an important role in tapetum development by regulating the expression of *DEFECTIVE IN TAPETAL DEVELOPMENT AND FUNCTION 1* (*TDF1*) in *Arabidopsis* [43]. Also, DYT1 is known to interact with Basic helix-loop-helix protein 89 (bHLH89) which is highly expressed in anthers and required for normal anther development and male fertility [44]. *TDF1* homolog (TRINITY_DN97604_c1_g7_i1) as well as *bHLH89* homolog (TRINITY_DN85771_c0_g1_i1) showed male-biased expression in *C. grandis* (Table 4; Additional file 8: Table S5)*.* Differential regulation of these genes related to stamen development explains the possible cause for early stamen arrest in female flowers of *C. grandis*.

**Table 4:** Digital Expression profile for genes in anther developmental pathway

Stage of stamen development	Genes	Expression pattern
Stamen Primordia Initiation	AG	Unbiased
	CLV1/CLV2	Unbiased/male-biased
	PI	Male-biased
	AP3	Unclear homolog
	JAG	Male-biased
Archesporial initiation	BAM1/BAM2	Unclear homolog
	SPL/NZZ	Unclear homolog
Tapetal Development	EMS1	Male-biased
	SERK1/2	Male-specific
	TPD1	Male-biased
	RPK2	Male-specific
	TDF	Male-biased
	DYT1	Unbiased
	bHLH89	Male-biased
Mature Pollen Formation	AMS	Male-biased
	MS1	Male-biased
	MS2	Male-biased
	MIA	Unbiased
	LAP3	Unbiased
	LAP5	Male-biased

According to recent reports from monoecious cucurbits like melon, cucumber, and watermelon, ethylene plays a major role in sex determination by inhibiting stamen development process [45–48]. We found that compared to male, GO:0009723 (response to ethylene) was enriched in female buds indicating a potential role of ethylene in sex determination of *C. grandis* (Additional file 9: Table S6).

### AgNO_3_ treatment on female plant releases the stamen inhibition

Female plants of *C. grandis* bear flowers with fused carpels and rudimentary stamens. Earlier, we have shown that foliar spray of 35 mM AgNO_3_ on the female plant of *C. grandis* promotes further development of the rudimentary stamens [14]. In the current study, gene expression profiles for early-staged Ag-H flower buds were compared with female buds (Table 3; Fig. 5c, g). Ag^+^ ions are known to inhibit responses to ethylene, a gaseous plant hormone [17]. Also, silver compounds have been shown to induce maleness by promoting stamen development in many monoecious and dioecious species [19–21]. No other inhibitors of ethylene biosynthesis or signaling could induce the stamen development in *Silene latifolia,* suggesting that ethylene signaling might not be the only pathway that gets affected upon application of silver thiosulphate [21]*.* In contrast to *Silene latifolia*, AVG (aminoethoxyvinylglycine), an inhibitor of ethylene-biosynthesis has been shown to induce male flowers in gynoecious muskmelon similar to silver compounds [49]. Considering the role of 1-aminocyclopropane-1-carboxylate synthase (ACS, an enzyme involved in ethylene biosynthesis) in sex determination of many other members of Cucurbitaceae, an ethylene-mediated effect of AgNO_3_ seems more likely to be involved in the modification of sex in *C. grandis* [50].

In our study, GO:0009723 (response to ethylene) and GO:0009873 (ethylene-activated signaling pathway) were enriched in female buds compared to Ag-H buds (Additional file 9: Table S6). Transcripts for genes such as *Ethylene-responsive transcription factors, ERF5* (TRINITY_DN102355_c3_g13_i1), *ERF17* (TRINITY_DN80749_c0_g6_i1), EF109 (TRINITY_DN87049_c0_g1_i1), EF102 (TRINITY_DN90257_c1_g2_i1), ERF99 (TRINITY_DN93821_c0_g1_i2), ERF60 (TRINITY_DN93262_c1_g6_i2) and *ERF78 (TRINITY_DN98503_c3_g1_i1)* were downregulated in Ag-H buds indicating impaired ethylene signalling (Additional file 8: Table S5). Additionally, qRT-PCR based expression pattern analysis for *ERF5*, *ERF17* and *EF102* genes clearly showed the suppression of ethylene responses by AgNO_3_ (Fig. 9)_._

Downregulation of ethylene signaling in Ag-H buds was correlated with the promotion of stamen growth. GO:0048655 (anther wall tapetum morphogenesis), GO:0048657 (anther wall tapetum cell differentiation), GO:0048658 (anther wall tapetum development) were seen to be enriched in early-staged Ag-H buds compared to female buds (Additional file 9: Table S6). *C. grandis* homologs of *MS1*, *MMD1* (TRINITY_DN109512_c4_g3_i1, TRINITY_DN108927_c0_g6_i1), *ZAT3* (TRINITY_DN108658_c0_g2_i1) and *AMS* (TRINITY_DN116105_c0_g2_i1) genes which play important roles in tapetum and pollen development of *Arabidopsis* flowers were upregulated upon AgNO_3_ treatment indicating promotion of stamen growth [51–56] (Additional file 8: Table S5; Fig. 7). *MYB35* (TRINITY_DN92649_c0_g7_i1), which was proposed as a putative sex-determining gene in *Asparagus* was also found to be upregulated in Ag-H buds [57] (Additional file 8: Table S5). Apart from that, gene ontology terms related to pollen wall assembly (GO:0010208), pollen exine formation (GO:0010584), sporopollenin biosynthetic process (GO:0080110), pollen development (GO:0009555) and pollen sperm cell differentiation (GO:0048235) were also enriched in Ag-H buds (Additional file 9: Table S6). Further, we noticed that *Ethylene-responsive transcription factors* (ERFs) were not affected in GyM-H buds as compared to female buds suggesting that stamen development in GyM-H flower buds might be regulated by some other mechanism evading ethylene signaling inhibition.

### Transcripts governing pollen fertility are depleted in GyM-H and Ag-H flower buds

*C. grandis* is one of the few species in which the presence of heteromorphic sex chromosomes is reported. The large Y-chromosome present in males might play a major role in sex determination. The GyM form of *C. grandis* included in the current study does not have Y-chromosome [14]. GyM-H flowers still develop full-sized stamens despite lacking Y-chromosome. Similarly, AgNO_3_ treatment induces stamen development in female plants having XX sex chromosomes. However, the pollens from GyM-H and Ag-H flowers buds were found to be sterile unlike the pollens from male buds [14]. Differential expression analysis revealed that gene ontology terms for pollen tube (GO:0090406), pollen germination (GO:0009846), regulation of pollen tube growth (GO:0080092), pollen tube growth (GO:0009860) and microsporogenesis (GO:0009556) were enriched in middle-staged male buds compared to middle-staged GyM-H buds (Additional file 9: Table S6).

*GAUTE* plays an important role in pollen tube wall biosynthesis in *Arabidopsis* [58]. TRINITY_DN111340_c1_g1_i6, which showed similarity with *GAUTE* was enriched in male buds compared to GyM-H buds. Unlike most other plant cell walls, pollen tube wall does not contain callose or cellulose. Pectin methylesterases (PMEs) have been shown to play a very important role in the growth of pollen tubes [59–61]. *PME4* (TRINITY_DN14239_c0_g1_i1), *PME37* (TRINITY_DN3663_c0_g1_i1) and *PPME1* (TRINITY_DN66415_c0_g1_i1, TRINITY_DN71598_c0_g2_i1) were downregulated in GyM-H buds compared to male buds (Additional file 8: Table S5). This could be a possible cause for pollens from GyM-H not forming pollen tubes. H Zhan, Y Zhong, Z Yang and H Xia [62] has shown that *IPMKB* (Inositol polyphosphate multikinase beta) is an important factor for pollen development. We have found that TRINITY_DN96290_c0_g3_i2 transcript matching to Arabidopsis *IPMKB* (*AtIpk2beta)* was downregulated in GyM-H compared to male buds. Earlier, several reports have demonstrated that *MALE STERILITY 1* (*MS1*) gene of *Arabidopsis* expresses in tapetal cells and plays an important role in pollen maturation [51, 54, 55]. *C. grandis* homolog of *MS1*, TRINITY_DN109512_c4_g3_i1 was expressed in a male-biased manner (Additional file 8: Table S5; Fig. 7). Similarly, homologs of genes important for pollen tube growth such as *CSLD1* (TRINITY_DN92683_c0_g1_i1), *CDPKO* (TRINITY_DN93671_c0_g1_i3), *NRX1* (TRINITY_DN106708_c1_g2_i3), *PTR52* (TRINITY_DN112735_c0_g14_i3; TRINITY_DN112735_c0_g3_i1), *TAF6* (TRINITY_DN96231_c1_g1_i2) and *CALS5* (TRINITY_DN113564_c1_g1_i1) were enriched in male [63–68] (Fig. 8, Additional file 8: Table S5). Genes involved in pollen exine formation such as *FACR2*/*MS2* (TRINITY_DN74585_c1_g5_i3), *EA6* (TRINITY_DN76274_c1_g1_i1), *C70A2/DEX2* (TRINITY_DN99059_c0_g1_i1) were also upregulated in male [69–71]. *EMS1* (TRINITY_DN89942_c0_g7_i1), *SERK1* (TRINITY_DN108624_c1_g7_i5), *JASON* (TRINITY_DN83440_c0_g1_i1), *RPK2* (TRINITY_DN113423_c0_g1_i4), which are essential for microsporogenesis and pollen maturation were observed to be expressed at significantly higher levels in middle-staged male buds compared to GyM-H buds. [72–75] (Additional file 8: Table S5; Fig. 7).

Expression profiling for homologs of *MS1*, *EMS1*, *DYT1, PME4, PME37, PPME1, CSLD1, CDPKO, and PTR52* was studied by qRT-PCR for all the tissue samples including middle-staged Ag-H buds (Figs. 7 and 8; Table 4). Transcripts for all these homologs were downregulated in Ag-H buds and GyM-H buds, suggesting a male-biased expression pattern implicating the reason for pollen sterility in Ag-H and GyM-H buds.

## Conclusions

De novo-assembled transcriptome developed from RNA-Seq of different sexual phenotypes has enabled identification of *C. grandis* homologs of many genes known to be involved in flower development in species such as *Arabidopsis*, melon, and cucumber. We found out that many genes involved in stamen initiation, tapetal development, and pollen maturation were downregulated in female buds compared to male buds. Interestingly, ethylene response-related genes were upregulated in female buds compared to male buds indicating a probable role of ethylene in stamen suppression similar to monoecious cucurbits such as melon and cucumber. We speculate that the Y-chromosome might express genes that inhibit ethylene signaling or suppress the carpel development, the site of ethylene production leading to the formation of stamens in male flowers. This was supported by the observation that AgNO_3_ treatment suppressed ethylene responses and induced stamen development in female flowers of *C. grandis*. However, the pollens produced by Ag-H flowers were sterile indicating a decisive role of Y-chromosome in determining maleness. In accordance with this, the transcripts involved in pollen maturation, pollen germination, and pollen tube elongation were depleted in middle-staged GyM-H buds compared to male buds. This could be because of the absence of Y-chromosome in GyM plant. Altogether, differentially expressed genes identified in this study could shed light on the probable mechanisms of sex determination, differentiation, and modification in *C. grandis.*

## Additional files

Additional file 1: Table S1. List of primers used in this study. (PDF 328 kb)

Additional file 2: De novo-assembled *Coccinia grandis* floral bud transcriptome. Available at https://doi.org/10.6084/m9.figshare.5220874.v1. (FASTA 4100 kb)

Additional file 3: Figure S1. ExN50 statistic for *C. grandis* flower de novo transcriptome assembly. (TIFF 1799 kb)

Additional file 4: Table S2. Distribution of percent length coverage for the top matching Swiss-Prot database entries. (PDF 7 kb)

Additional File 5: Table S3. *Coccinia grandis* flower bud transcriptome metrics calculated using TransRate. Protein-coding primary transcripts of *Cucumis sativus* were chosen as reference. (PDF 10 kb)

Additional file 6: Table S4. Annotation report for the de novo-assembled *C. grandis* flower bud transcriptome generated using Trinotate. Available at https://doi.org/10.6084/m9.figshare.5217652.v1. (XLSX 5870 kb)

Additional file 7: Figure S2. Pairwise comparisons of transcript abundance. MA plots showing average log fold change (logFC) vs average log of counts across replicates. Volcano plots showing differentially expressed transcripts in relation to FDR (False discovery rate). Features found DE at FDR <0.05 are colored red. Features with *P*-values at most 1e-3 and at least 2^2 fold change are differentially expressed. (TIFF 5988 kb)

Additional file 8: Table S5. Expression matrix of transcripts with P-values at most 1e-3 and at least 2^2 fold differential expression. (XLS 7379 kb)

Additional file 9: Table S6. Gene ontology (GO) enrichment analysis for differentially expressed transcripts in each pairwise comparison. (XLSX 1509 kb)

Additional file 10: Figure S3. Transcript clusters extracted from the hierarchical clustering with R. X-axis: samples; y-axis: median-centered log_2_(FPKM). Grey lines, individual transcripts; blue line, average expression values per cluster. (TIFF 5037 kb)

## Abbreviations

Ag-H flower buds: Hermaphrodite flowers from a plant treated with silver nitrate
AVG: Aminoethoxyvinylglycine
BUSCO: Benchmarking Universal Single-Copy Orthologs
CRB-BLAST: Conditional Reciprocal Best BLAST
edgeR: Empirical Analysis of Digital Gene Expression Data in R
eggNOG: evolutionary genealogy of genes: Non-supervised Orthologous Groups
FPKM: Fragments per kb per million reads
GO: Gene ontology
GyM: Gynomonoecious
GyM-H flower buds: Hermaphrodite flowers from a gynomonoecious plant
HMMER: Tool used for searching sequence databases for sequence homologs
KEGG: Kyoto encyclopedia of genes and genomes
NGS: Next-generation sequencing
Nr: Non-redundant protein database
PFAM: database of protein families, each represented by multiple sequence alignments and hidden Markov models (HMMs)
qRT-PCR: Quantitative real-time PCR
RIN: RNA integrity number
RSEM: RNA-Seq by Expectation-Maximization
Swiss-Prot: Annotated protein sequence database
TrEMBL: Computer-annotated supplement to Swiss-Prot database
