# Supplementary material for: De novo transcriptome assembly from flower buds of dioecious, gynomonoecious and chemically masculinized female Coccinia grandis reveals genes associated with sex expression and modification
Source: BMC Plant Biol. 2017 Dec 12;17:241. doi: 10.1186/s12870-017-1187-z (PMC5727884; doi:10.1186/s12870-017-1187-z)
Supplement: Supplementary file 1 — List of primers used in this study. (PDF 328 kb) [file 12870_2017_1187_MOESM1_ESM.pdf]

Table S1 List of primers used in this study.

Primer name	Sequence (5′ -> 3′)
DEX1_qF	CTATGTCTGTTCCCCTGCCCTG
DEX1_qR	GACCAGACTTTTCAATCACCCATG
CER3_qF	TCCCAACACTTAGACTCTCAACAC
CER3_qR	GGAAAGGTTCACTATTGGGCGTG
EMS1_qF	TCCGTCAAGTTGTTACACACCAC
EMS1_qR	TGCTCCACTCCTCTGTCTCTCC
TPD1_qF	TGCAACTGAAATGACACTCGCC
TPD1_qR	ACCAATCTAACAGGCCACACAC
ZAT3_qF	ACGACGGAAACTAAGCGGCG
ZAT3_qR	GCGCCGAAGATCACTCCTCC
DYT1_qF	GAAGCGGAAAGGGGCATTCAC
DYT1_qR	TGACGTGCAAAAGAGCCCATTG
AMS_qF2	GGGAGCTCTGGATTGTCTTCGGG
AMS_qR2	TCTCCGGCTTCCTTTATCCCGC
MMD1_qF2	TTCAGTGTGATCAATTGTGTCTCGC
MMD1_qR2	TTGGAAGGTGGGGCTACAGATTC
MS1_qF2	CGACCGAGCAACAGGTGAAAC
MS1_qR2	TCCTTCAGCTCATCAATGGTGGC
FERONIA_qF	AGATCCACCTCCCACTACACCC
FERONIA_qR	ACCGCAACTCCAACCACAACAG
EIL1_qF	GAGAACGCTAACTTTCCACGCC
EIL1_qR	TGGAAACGAGGAATGGTGGCC
SHT_qF	CAAGGTTGTGGCTGCCGATG
SHT_qR	GTTGTCGTCGTTGTTGCGGG
PME4_qF	TGTTGGAATCTTGAACCGCTCT
PME4_qR	ACAACCAACAGAACACCGTCAC
PME37_qF	CCAAAAGAAGTCTCGGGGCTGG

PME37_qR	CCGGTCTTGCGTATTGCTTCCA
PPME1_qF	GTATTTTACGCGGGCACTCGAC
PPME1_qR	GCTGGAACGACATGAAGCATCC
ERF5_qF	CATCACTATCCCAAACCGTCGC
ERF5_qR	GGGAAATGGGAAACAAAGCGGT
ERF17_qF	TCGTCCCCTCACAAATCCTCCT
ERF17_qR	CGCACTCCTTTGTACCGTGAAT
EF102_qF	AGTCGTAGAGGTTGTTGAGGCG
EF102_qR	CCAAACCATCGACCAAGACGAC
CSLD1_qF	CGAGGTTTTCGCCACTCCAAAG
CSLD1_qR	ACCAAAGAGAAAAGAAGCAGGTGGA
CDPKO_qF	AGAGGAGCAGAGACATTAAGCCC
CDPKO_qR	TCGGAGAGAATGGAAGACGCAA
PTR52_qF	ATCCTCCAAGTTTCCGACGAGG
PTR52_qR	ACCTTCTCCCTTCCACAGCTTC
