# Supplementary figures and images for: De novo transcriptome assembly from flower buds of dioecious, gynomonoecious and chemically masculinized female Coccinia grandis reveals genes associated with sex expression and modification
Source: BMC Plant Biol. 2017 Dec 12;17:241. doi: 10.1186/s12870-017-1187-z (PMC5727884; doi:10.1186/s12870-017-1187-z)

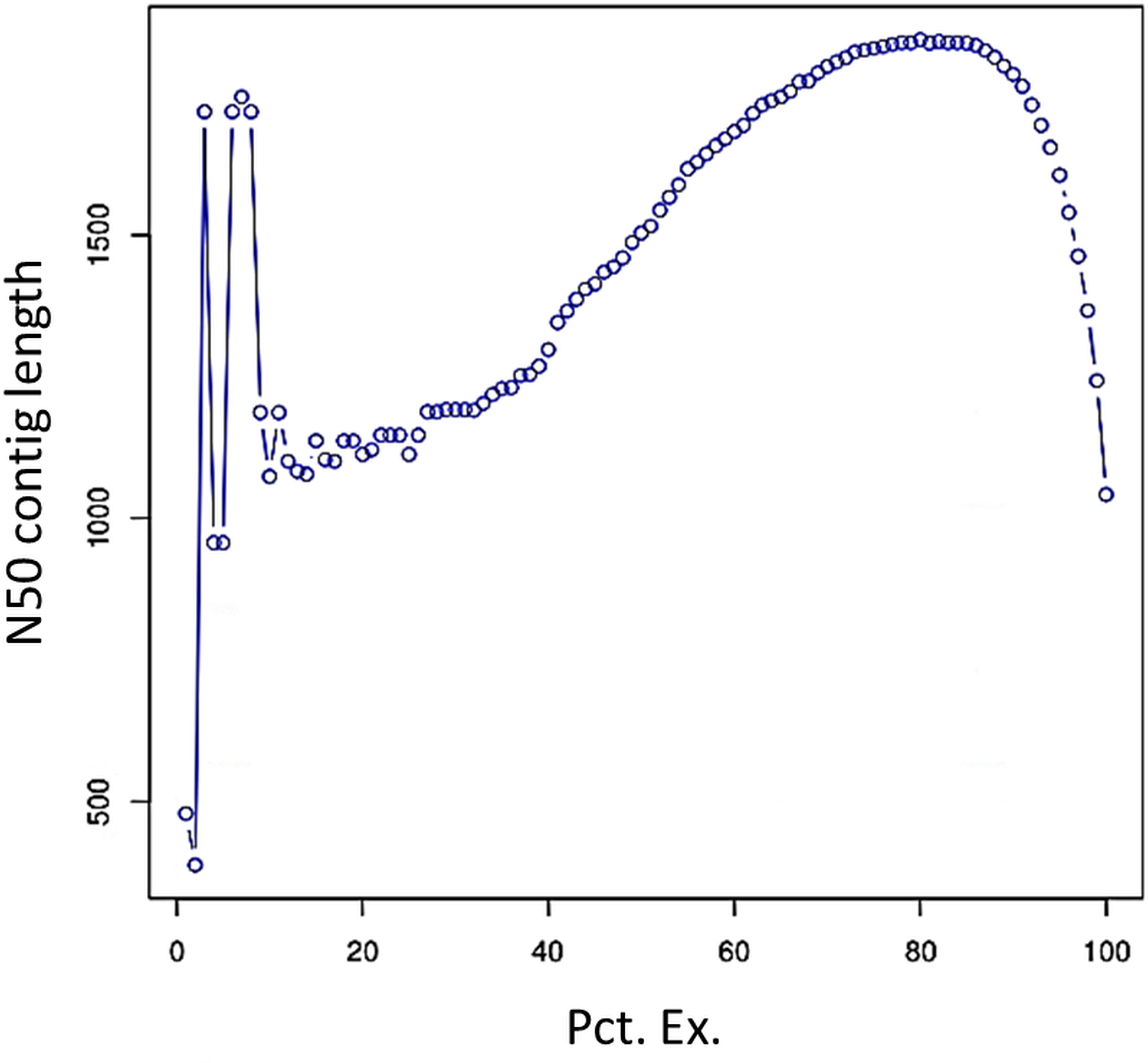

Supplement: Supplementary file 3 — ExN50 statistic for C. grandis flower de novo transcriptome assembly. (TIFF 1799 kb) [file 12870_2017_1187_MOESM3_ESM.tif]

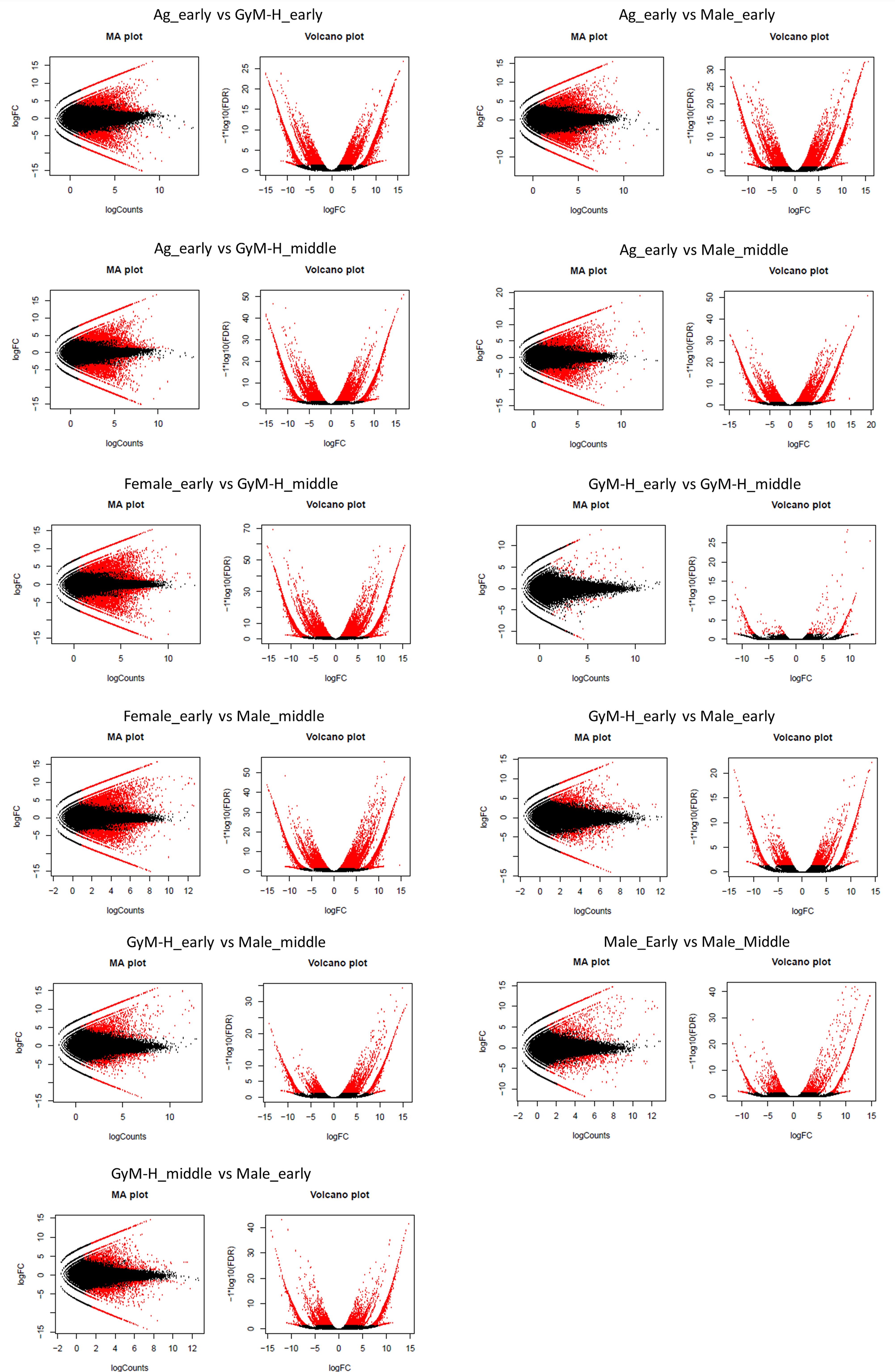

Supplement: Supplementary file 7 — Pairwise comparisons of transcript abundance. MA plots showing average log fold change (logFC) vs average log of counts across replicates. Volcano plots showing differentially expressed transcripts in relation to FDR (False discovery rate). Features found DE at FDR <0.05 are colored red. Features with P-values at most 1e-3 and at least 2^2 fold change are differentially expressed. (TIFF 5988 kb) [file 12870_2017_1187_MOESM7_ESM.tif]

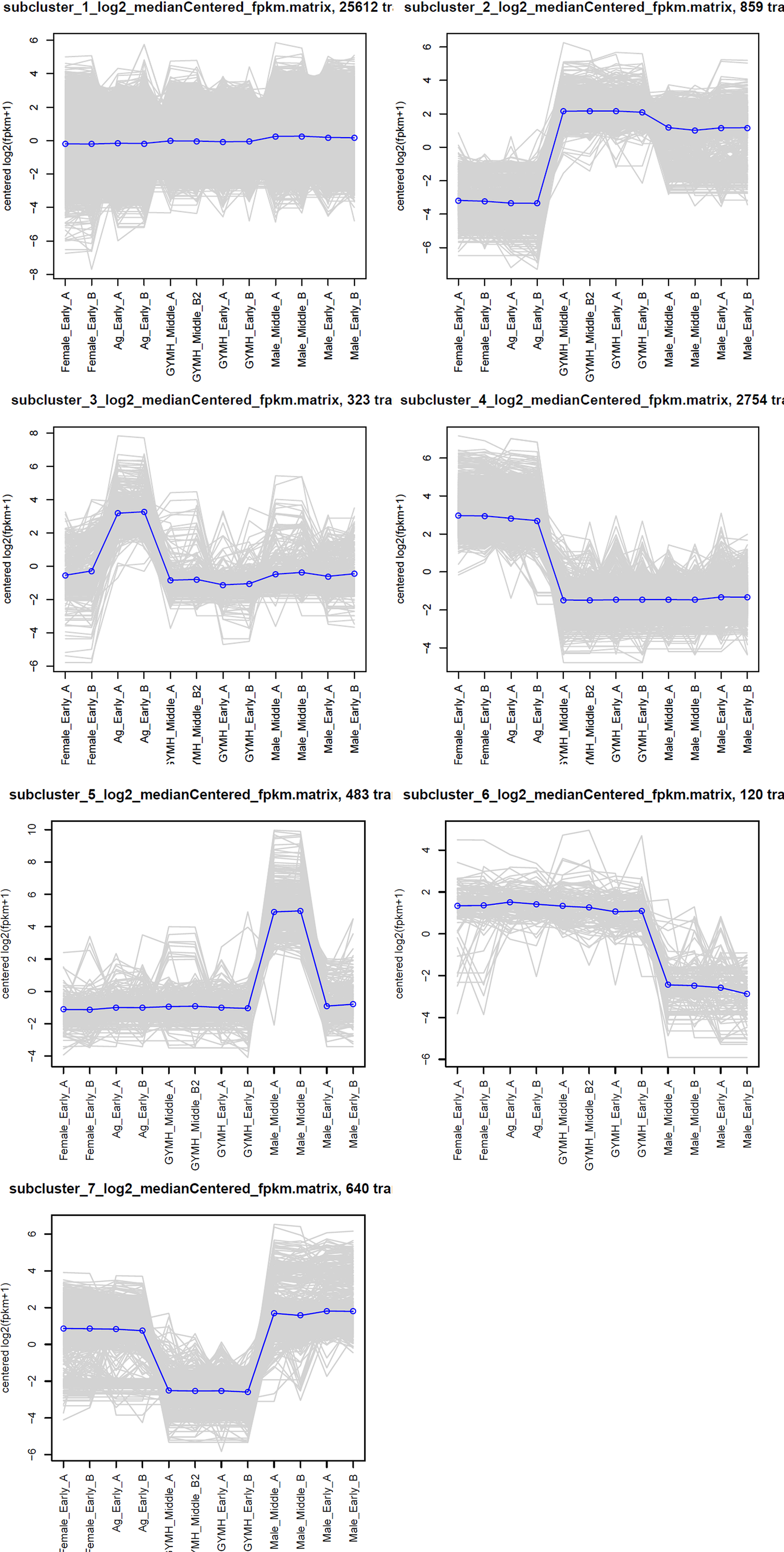

Supplement: Supplementary file 10 — Transcript clusters extracted from the hierarchical clustering with R. X-axis: samples; y-axis: median-centered log2(FPKM). Grey lines, individual transcripts; blue line, average expression values per cluster. (TIFF 5037 kb) [file 12870_2017_1187_MOESM10_ESM.tif]
