# Supplementary material for: De novo transcriptome assembly from flower buds of dioecious, gynomonoecious and chemically masculinized female Coccinia grandis reveals genes associated with sex expression and modification
Source: BMC Plant Biol. 2017 Dec 12;17:241. doi: 10.1186/s12870-017-1187-z (PMC5727884; doi:10.1186/s12870-017-1187-z)
Supplement: Supplementary file 4 — Distribution of percent length coverage for the top matching Swiss-Prot database entries. (PDF 7 kb) [file 12870_2017_1187_MOESM4_ESM.pdf]

**Table S2** Distribution of percent length coverage for the top matching SwissProt database entries.

| #hit_pct_cov_bin | count_in_bin | >bin_below |
|---|---|---|
| 100 | 5680 | 5680 |
| 90 | 1838 | 7518 |
| 80 | 1398 | 8916 |
| 70 | 1473 | 10389 |
| 60 | 1926 | 12315 |
| 50 | 2765 | 15080 |
| 40 | 5137 | 20217 |
| 30 | 10818 | 31035 |
| 20 | 28691 | 59726 |
| 10 | 46993 | 106719 |
