# Supplementary material for: De novo transcriptome assembly from flower buds of dioecious, gynomonoecious and chemically masculinized female Coccinia grandis reveals genes associated with sex expression and modification
Source: BMC Plant Biol. 2017 Dec 12;17:241. doi: 10.1186/s12870-017-1187-z (PMC5727884; doi:10.1186/s12870-017-1187-z)
Supplement: Supplementary file 5 — Coccinia grandis flower bud transcriptome metrics calculated using TransRate. Protein-coding primary transcripts of Cucumis sativus were chosen as reference. (PDF 10 kb) [file 12870_2017_1187_MOESM5_ESM.pdf]

Table S3 *Coccinia grandis* flower bud transcriptome metrics calculated using TransRate. Protein-coding primary transcripts of *Cucumis sativus* were chosen as reference.

**TransRate contig metrics**			
**Contig metrics**		**Comparative metrics**	
n seqs	467233	CRBB hits	115567
smallest	201	n contigs with CRBB	115567
largest	17089	p contigs with CRBB	0.25
n bases	2.83E+08	rbh per reference	5.37
mean len	606.45	n refs with CRBB	18039
n under 200	0	p refs with CRBB	0.84
n over 1k	68072	cov25	17607
n over 10k	44	p cov25	0.82
n with orf	70688	cov50	16890
mean orf percent	54.73	p cov50	0.79
n90	260	cov75	15744
n70	441	p cov75	0.73
n50	881	cov85	14934
n30	1713	p cov85	0.69
n10	3162	cov95	13430
gc	0.39	p cov95	0.62
bases n	0	reference coverage	0.84
proportion n	0		
